# Midline Destructive Lesions: Differentiating Granulomatosis With Polyangiitis From Cocaine-Induced Perforations

**DOI:** 10.7759/cureus.83425

**Published:** 2025-05-03

**Authors:** Khalid Seedahmed, Malaz Kamaleldin Omer, Mohammed Osman

**Affiliations:** 1 Ear, Nose, and Throat (ENT), Sligo University Hospital, Sligo, IRL; 2 Internal Medicine, Sudan Medical Specialization Board, Khartoum, SDN

**Keywords:** c-anca, case report, cocaine-induced midline destructive lesions, granulomatosis with polyangiitis, nasal perforation, palatal perforation

## Abstract

Granulomatosis with polyangiitis (GPA) is a rare autoimmune vasculitis that may present with midline destructive lesions, often overlapping with conditions such as cocaine-induced midline destructive lesions (CIMDL).

A 56-year-old man with a history of significant cocaine use (1 gram twice a week for three years, abstinent for five years) presented with chronic nasal congestion and epistaxis. He developed a large nasal septal perforation and later a palatal perforation. Laboratory workup revealed positive cytoplasmic antineutrophil cytoplasmic antibody (C-ANCA) with elevated proteinase 3 (PR3) levels, supporting the diagnosis of GPA. Despite an initial lack of granulomatous inflammation on biopsy, the patient's clinical presentation and positive response to immunosuppressive therapy confirmed GPA as the correct diagnosis.

This case highlights the complexity of diagnosing GPA in a patient with a history of cocaine use. The positive C-ANCA result, systemic symptoms, and response to immunosuppressive treatment were key in establishing the diagnosis. It underscores the importance of considering GPA in the differential diagnosis of midline destructive lesions, even in the context of a history of cocaine use, and the need for timely and multidisciplinary management.

## Introduction

The palate forms the roof of the oral cavity, separating it from the nasal cavity. It comprises two parts: an anterior hard palate, which is immobile and bony, and the posterior soft palate, which is mobile and lacks bone [1]. Palatal lesions can vary in severity, from simple ulcers to more severe conditions, such as perforations.

Palate perforation is an uncommon clinical condition characterized by loss of the tissue components of the palate, affecting its entire thickness, whether soft or hard. This results in communication between the oral cavity and the nasal or nasopharyngeal space [2]. The etiology of palatal perforation can be classified into infectious causes, including tuberculosis, syphilis, leprosy, and fungal infections, as well as non-infectious factors like developmental abnormalities, neoplasms, drug use, autoimmune diseases, and iatrogenic factors [3]. Rare causes include rhinoliths and psychological issues.

Granulomatosis with polyangiitis (GPA), formerly known as Wegener's granulomatosis, is a rare, multisystem, necrotizing vasculitis characterized by non-caseating granulomas. It primarily affects small- to medium-sized arteries, capillaries, and veins [4,5]. The condition was first described by Peter McBride in 1897. In 1936, Dr. Friedrich Wegener, a German pathologist, reported three cases characterized by a septic course, arteritis, glomerulonephritis, and granulomatosis of the nasopharynx and larynx [6,7]. The disease is most commonly seen among Caucasians, typically affecting individuals between the fifth and seventh decades of life, with a slight male predominance [8-10].

Cocaine is a widely used alkaloid stimulant derived from the leaves of Erythroxylon coca, which emerged in the 1970s. It is the second most frequently used illegal drug in England and Wales, and in 2016, it was estimated that around 17 million adults have used cocaine at least once in their lifetime [11,12]. Two primary forms of cocaine exist: cocaine hydrochloride - a white powder typically snorted or injected, and crack cocaine - a freebase form that is smoked, affecting the nasal cavity commonly [13-15]. Both forms have a rapid onset of action, within seconds to two minutes, and peak effects occur within 15-60 minutes.

Cocaine exerts its effects by acting as a local anesthetic, blocking voltage-gated sodium channels to prevent nerve signal transmission, and causing local vasoconstriction, which leads to mucosal irritation [10]. Chronic use can result in ischemic osseocartilaginous necrosis, leading to the perforation of midline structures, a condition known as cocaine-induced midline destructive lesions (CIMDLs). Cocaine's ability to induce apoptosis depends on the dose and duration of exposure.

## Case presentation

Patient background

A 56-year-old man who is an active smoker with a history of significant cocaine use (approximately 1 gram twice a week for three years), though he reported abstinence for the past five years, he has a longstanding history of asthma, nasal congestion, and intermittent epistaxis. Additionally, he reported a productive cough with green sputum and generalized fatigue.

The patient also reported intermittent pins-and-needles sensations in the extremities, raising suspicion of peripheral neuropathy. However, neurological examination revealed normal power, tone, reflexes, and sensory responses in all limbs, with normal plantar reflexes bilaterally. The patient has been referred to the neurology team for further evaluation. Chest examination was also unremarkable, showing no evidence of crepitations or wheezing. Upon ear, nose, and throat (ENT) examination, a large septal perforation with significant destruction of the cartilaginous and bony components of the nasal septum was identified.

Concurrently, the patient sought medical evaluation for chronic anemia with low serum folate and iron levels. Gastroscopy and colonoscopy were performed, revealing chronic gastrointestinal disease characterized by mild to moderate reactive gastritis and intestinal metaplasia in gastric biopsies. No abnormalities were identified in the colonoscopic examination. He had a reassuring CT scan of the thorax, abdomen, and pelvis.

Laboratory and histopathological findings

**Figure 1:**
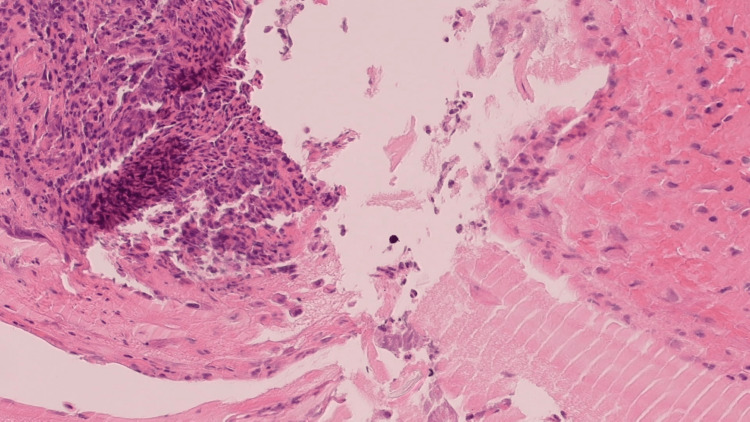
Squamous and respiratory mucosa with acute and chronic inflammation, granulation tissue, and histiocytes, H&E, medium power

**Figure 2:**
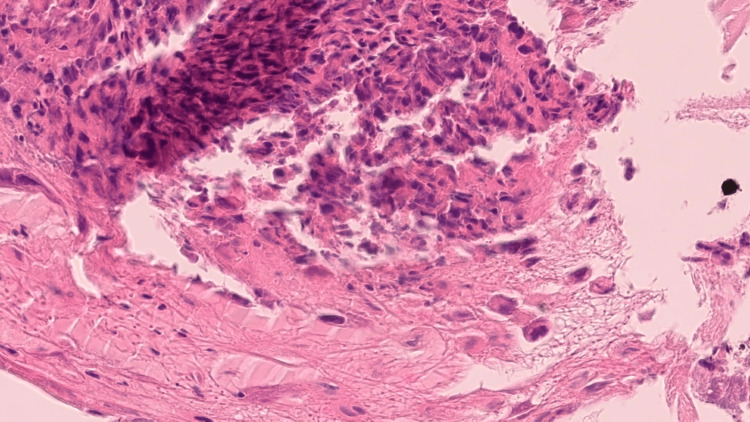
Squamous mucosa with granulation tissue, H&E, medium power

Serological investigations revealed a positive cytoplasmic antineutrophil cytoplasmic antibody (C-ANCA); the proteinase 3 (PR3) level was 11, while anti-myeloperoxidase (MPO) was normal. CRP was 46, and urine dipstick analysis was normal, with no evidence of leukocytes, nitrates, protein, or blood. Biopsies taken from the edges of the nasal perforation demonstrated acute inflammation with fibrinoid debris, showing no evidence of granulomatous inflammation or vasculitis (Figures 1-2).

Late development of palatal perforation

**Figure 3:**
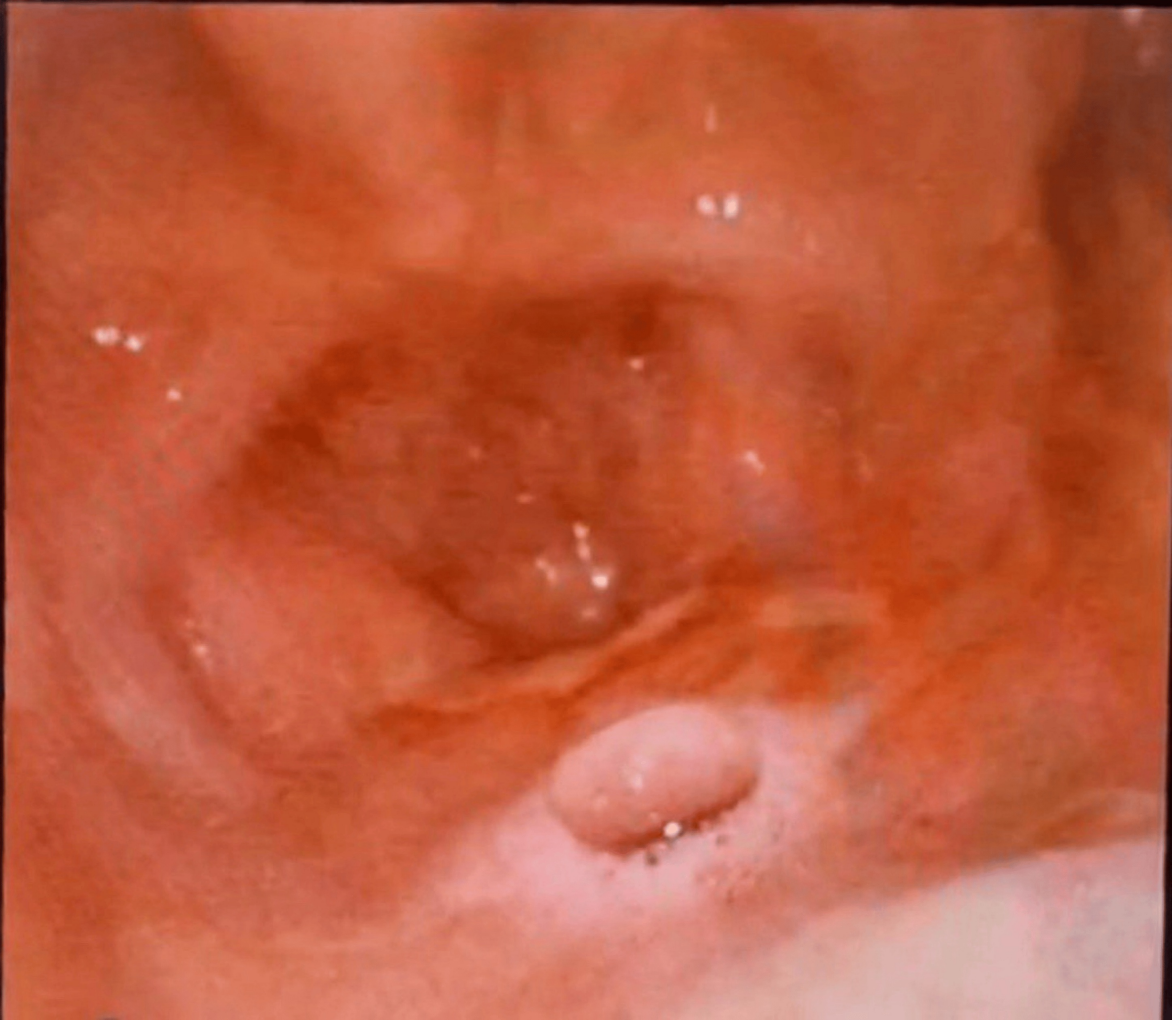
The nasoendoscopic view shows the palatal hole with an air bubble on the floor of the nasal cavity with no septum in the middle and the postnasal space

**Figure 4:**
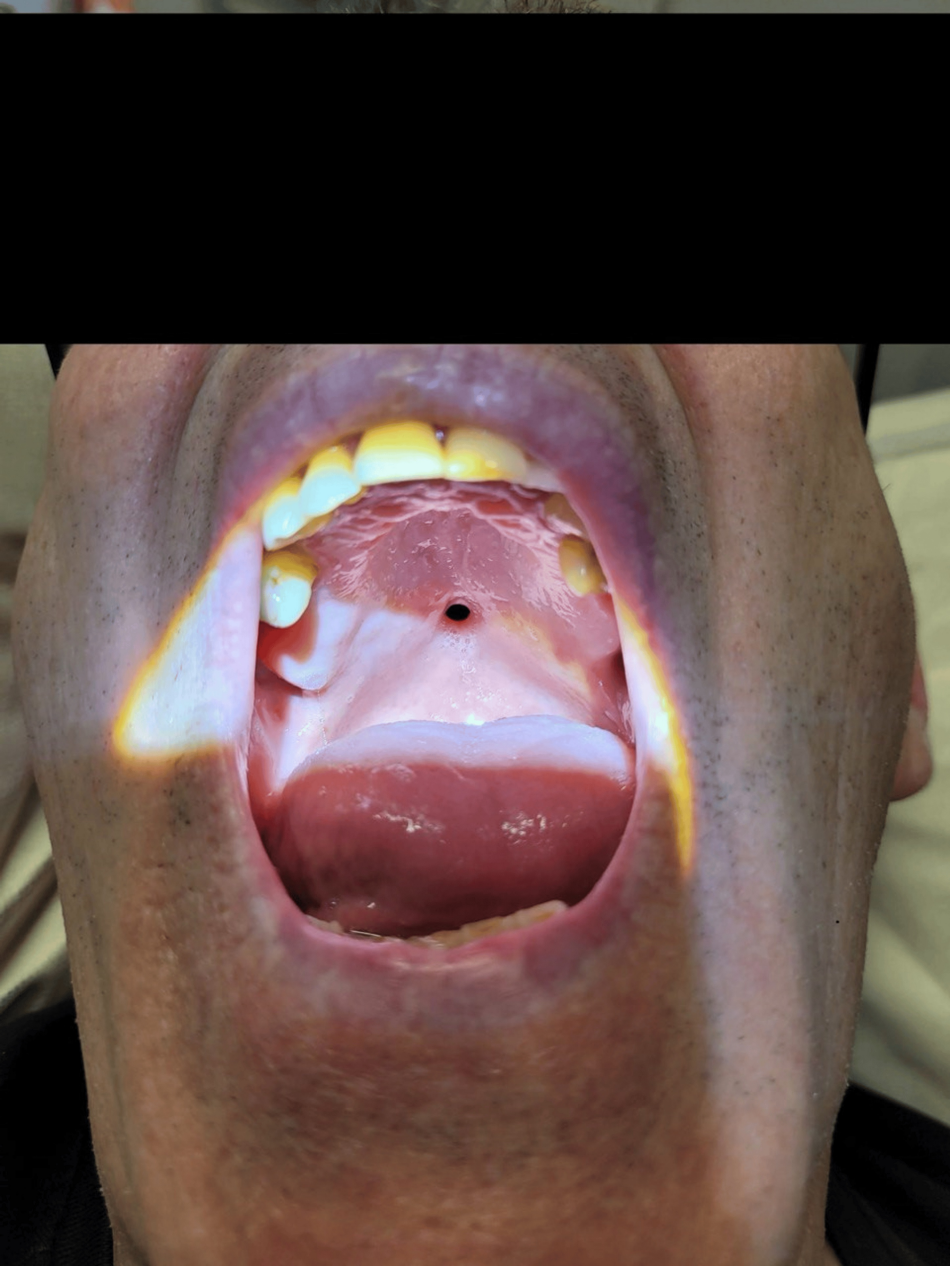
Perforation at the junction of the soft and hard parts of the palate after two days of reporting food regurgitation

**Figure 5:**
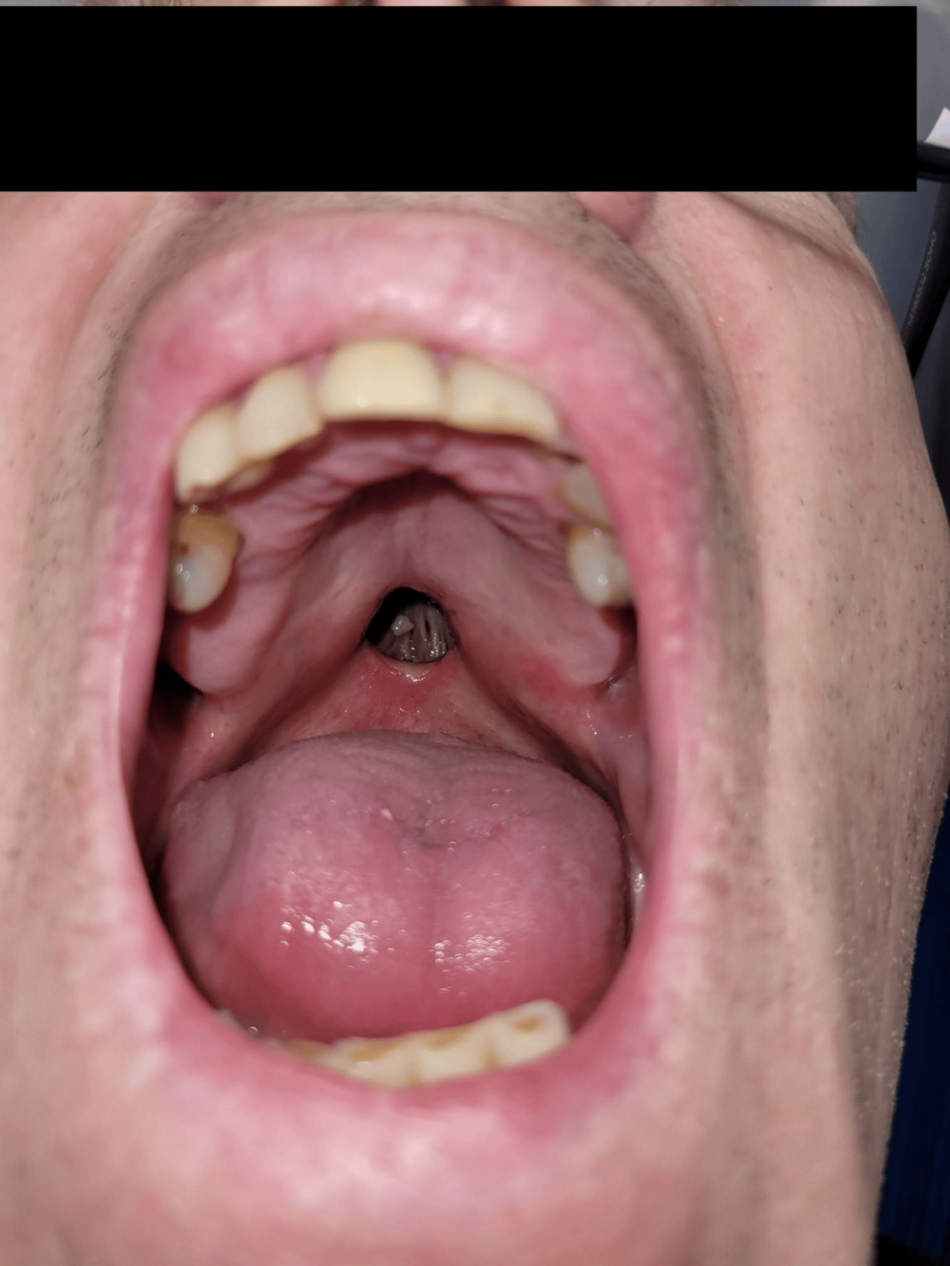
The perforation increased in size after two months, indicating regurgitation and rhinolalia aperta

During a gastroenterology follow-up visit, the patient reported experiencing nasal regurgitation of food. An ENT examination revealed a new finding of a small palatal perforation (Figures 3-4), which progressively enlarged over time (Figure 5). This resulted in severe rhinolalia aperta and significant communication difficulties, causing considerable frustration and a diminished quality of life.

Management plan

The patient was initiated on oral prednisolone (initially 40 mg/day, tapered gradually) and methotrexate (15 mg/week), along with folic acid supplementation and antibiotics. This immunosuppressive regimen has been continued for four months to date, under close monitoring by the rheumatology team.

Clinical remission of GPA was evaluated based on symptomatic improvement and stabilization of the palatal defect. Laboratory parameters - including CRP, erythrocyte sedimentation rate (ESR), and PR3-ANCA levels - demonstrated favorable trends on serial follow-up. A repeat biopsy was avoided to prevent further enlargement of the palatal fistula.

**Figure 6:**
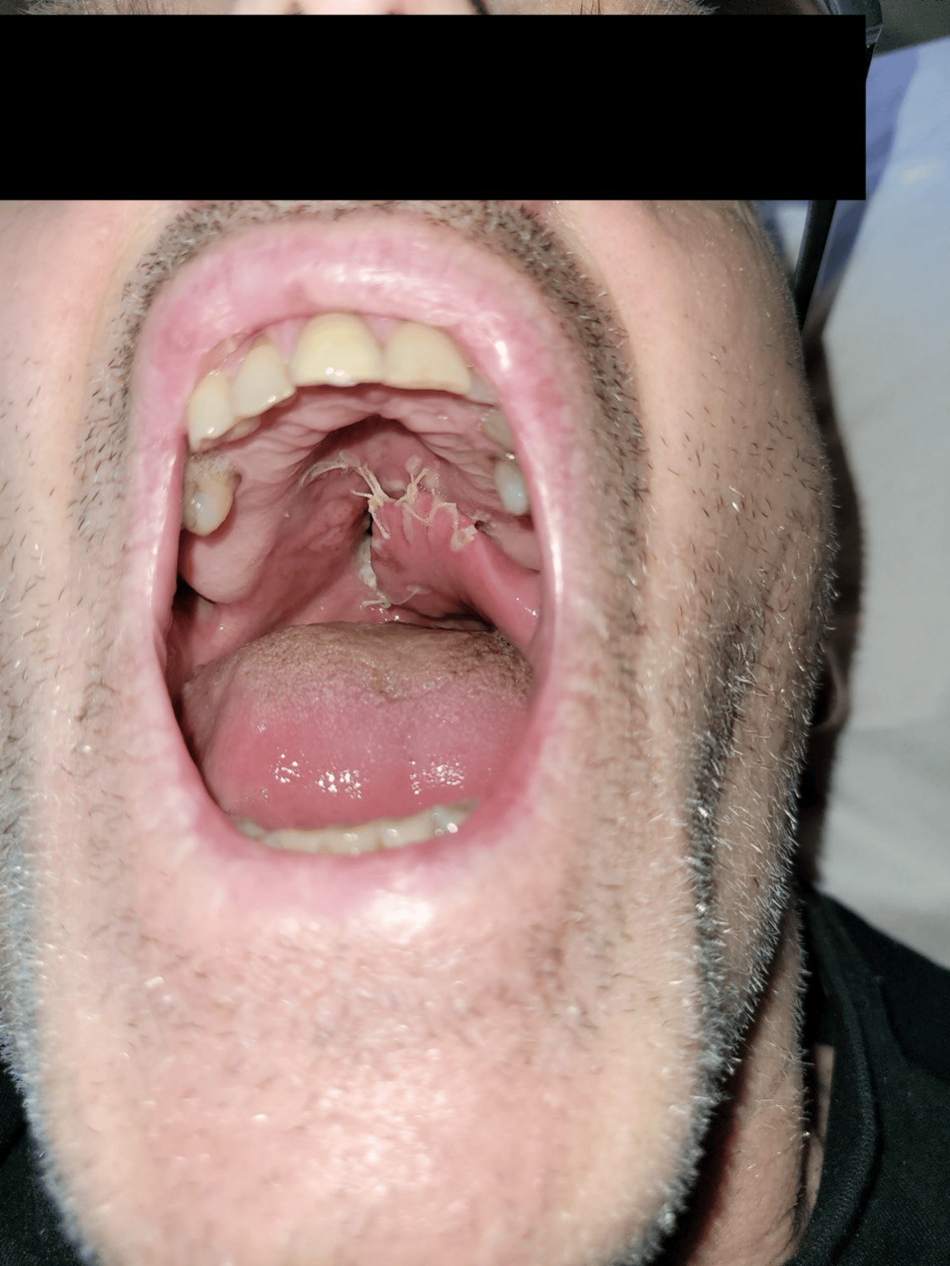
Left buccinator pedicled flap raised to cover the palatal defect as a stage one procedure, with a near-future planned second-stage step to release the pedicle

After a one-month assessment by the maxillofacial team to confirm disease stability, a staged reconstructive approach was initiated. A left-sided pedicled buccinator flap was raised to cover the palatal defect (Figure 6).

Recently, the patient underwent a revision procedure due to dehiscence of the initial flap. During the surgery, the flap was revised, a palatal flap from the posterior aspect was advanced to cover the fistula, and the left buccinator pedicle was divided. Additionally, another buccinator flap was raised from the right side to further support the repair.

## Discussion

Midline destructive lesions (MDLs) are recognized as outcomes of various disease processes, including autoimmune vasculitis (e.g., GPA), infections, malignancies, and drug-induced vasculitis [16,17]. In this case, the patient's clinical presentation, characterized by a significant history of cocaine use, nasal symptoms, and palatal perforation, poses a diagnostic challenge in distinguishing between GPA and CIMDLs. Both conditions can lead to severe MDLs, positive ANCA serology, and histopathologic evidence of vasculitis, complicating diagnosis. A comprehensive clinical history is essential, although patients may not disclose drug abuse. Nevertheless, key distinctions can be identified based on systemic involvement, disease chronicity, the type of ANCA antibodies present, and response to treatment.

Sinonasal involvement is the most common manifestation of GPA, affecting up to 85% of patients [18,19]. Over 25% of patients present with only sinonasal symptoms, such as nasal obstruction, rhinitis, and epistaxis, which are frequently misdiagnosed as chronic rhinosinusitis. In some cases, these symptoms may persist for months or even years before a correct diagnosis is reached. Additionally, tracheobronchial involvement occurs in 13.6-55% of GPA cases, with subglottic stenosis being the most common manifestation [20]. Wheezing associated with subglottic stenosis may be incorrectly diagnosed as asthma, especially in the absence of other organ involvement. Calabrese et al. reported a case of GPA that was misdiagnosed as severe asthma for eight years before the correct diagnosis was made [21].

Up to 67% of patients with GPA experience peripheral nervous system involvement, either as polyneuropathy or mononeuritis multiplex, due to vasculitis of the vasa nervorum [22]. This can account for the patient's history of peripheral numbness. Other neurological manifestations of GPA include cranial neuropathies, cerebrovascular accidents, headaches, diabetes insipidus, and, in rare cases, central nervous system masses [23]. Furthermore, the patient's history of chronic fatigue can be attributed to the musculoskeletal manifestations of GPA, including arthralgia, myalgia, and arthritis, which affect approximately 25% of GPA patients [18].

The term "cocaine-induced pseudovasculitis" has been used to describe the clinical and serological features observed in patients with a history of cocaine use. It remains uncertain whether cocaine itself is the primary cause of the pathological changes or if levamisole, an anti-helminthic veterinary drug often mixed with cocaine, is the main trigger [24]. Levamisole acts synergistically with cocaine to enhance its dopaminergic effect, which in turn promotes addiction [25-27]. However, both substances have been independently linked to vasculitis, indicating that both may contribute to disease development [28-30]. The most common manifestations include MDLs and cutaneous vasculitis, frequently presenting as a retiform purpuric rash on the face and lower limbs [31,32].

A prospective study by Trimarchi et al. [33] reported distinct patterns of perforations in CIMDL and GPA. In CIMDL, perforations are commonly observed in the nasal septum, with necrosis extending from the septum to the nasal walls. Erosion of the inferior turbinates occurs in 75% of CIMDL patients, while middle turbinate involvement is observed in 62.5%. Destruction of other midfacial structures, such as the lateral nasal wall, medial maxillary sinus wall, and hard palate, is commonly seen, with the degree of septal perforation correlating to the extent of damage to other structures. In contrast, GPA typically results in nasal septum perforation, but the involvement of other midline structures, including the turbinates and palate, is uncommon, with only 12.5% of patients exhibiting erosion beyond the septum.

Although histopathological examination is crucial for distinguishing GPA from CIMDL, the findings often overlap. In GPA, extravascular changes characterized by giant cells and microscopic foci of deeply located necrosis are considered diagnostic [33-37]. However, biopsy specimens are definitive in only about 50% of patients with GPA. In contrast, CIMDL commonly presents with vascular changes, including microabscesses in the venule wall, leukocytoclastic vasculitis, and thrombosis of venules and arterioles, which are marked by fibrin deposition or organizing intravascular granulation tissue [33]. These features can also occur in GPA, raising the possibility of misinterpreting CIMDL biopsies as GPA. Furthermore, identifying apoptotic cells through in situ Terminal deoxynucleotidyl transferase-mediated dUTP nick end labeling (TUNEL) assays can act as a distinguishing characteristic since apoptosis is a hallmark of CIMDL but is rarely seen in GPA [38].

In our patient, the positive C-ANCA result, specifically targeting PR3, supported our diagnosis of GPA despite the absence of confirmatory histological evidence from the biopsy. C-ANCA is elevated in 90% of patients with active generalized GPA and serves as a reliable marker for disease activity [39-43]. However, a normal C-ANCA result does not exclude GPA, particularly in cases of limited disease [44].

In contrast, dual positivity for PR3 and MPO-ANCA is considered pathognomonic for CIMDL [45]. Additionally, the presence of anti-human neutrophil elastase (HNE) antibodies, which produce a perinuclear (P-ANCA) staining pattern and are found in up to 83% of CIMDL patients, has emerged as a key feature distinguishing CIMDL from GPA [29]. However, the limited availability of HNE-ANCA testing in routine clinical practice limits its use as a diagnostic tool in many centers.

While ANCA positivity in GPA is strongly associated with autoimmune mechanisms, the exact cause of ANCA production in CIMDL remains unclear. It may result from polyclonal B-cell activation induced by cocaine or its adulterants or from chronic *Staphylococcus aureus *nasal colonization, which may develop due to mucosal damage caused by cocaine-induced ischemia. *S. aureus *releases potent superantigens that activate T and B cells, bypassing the normal antigen-specific immune response [46].

GPA is typically treated with systemic glucocorticoids and immunosuppressive therapies such as cyclophosphamide. In cases of limited disease, oral methotrexate can serve as an effective alternative. For refractory cases, biologic therapies such as rituximab have been utilized with variable success [47]. In contrast, CIMDL requires cessation of cocaine use as the cornerstone of treatment [48-50]. While immunosuppressive therapies such as corticosteroids and cyclophosphamide have been applied in some cocaine-induced vasculitis cases, particularly with renal involvement, their effectiveness depends on the patient's discontinuation of cocaine [51,52].

## Conclusions

This case highlights the diagnostic challenges in distinguishing GPA presenting with MDLs in the context of prior cocaine abuse. Despite the absence of granulomatous inflammation on biopsy, the patient’s five years of cocaine abstinence, the overall clinical picture, positive ANCA-PR3, and favorable response to treatment were crucial in confirming the diagnosis of GPA. This case underscores the importance of a multidisciplinary approach to managing such complex cases and emphasizes the need for early recognition of GPA to initiate prompt and effective treatment, ultimately improving patient outcomes.
